# Decision aids for cancer survivors’ engagement with survivorship care services after primary treatment: a systematic review

**DOI:** 10.1007/s11764-022-01230-y

**Published:** 2022-07-07

**Authors:** Yu Ke, Hanzhang Zhou, Raymond Javan Chan, Alexandre Chan

**Affiliations:** 1 Department of Pharmacy, National University of Singapore, Singapore, Singapore; 2 Caring Futures Institute, College of Nursing and Health Sciences, Flinders University, Bedford Park, SA5042 Australia; 3 School of Nursing, Queensland University of Technology, Kelvin Grove, Australia; 4 Princess Alexandra Hospital, Metro South Hospital and Health Services, Woolloongabba, QLD Australia; 5 Department of Clinical Pharmacy Practice, University of California Irvine, Irvine, CA USA

**Keywords:** Cancer, Oncology, Survivorship, Decision aids, Decisional support tool, Shared decision making

## Abstract

**Purpose:**

To elucidate existing decision aids (DAs) in supporting cancer survivors’ decisions to engage in cancer survivorship care services after primary treatment. Secondary objectives are to assess the DA acceptability, impact of DAs, and implementation barriers.

**Methods:**

Databases (PubMed, Embase, PsycINFO, CINAHL) were searched to collect publications from inception through September 2021. Studies describing the development or evaluation of DAs used for survivorship care services after primary cancer treatment were included. Article selection and critical appraisal were conducted independently by two authors.

**Results:**

We included 16 studies that described 13 DAs and addressed multiple survivorship care domains: prevention of recurrence/new cancers in Hodgkin lymphoma survivors and breast cancer gene mutation carriers, family building options, health insurance plans, health promotion (substance use behavior, cardiovascular disease risk reduction), advanced care planning, and post-treatment follow-up intensity. The electronic format was used to design most DAs for self-administration. The content presentation covered decisional context, options, and value clarification exercises. DAs were acceptable and associated with higher knowledge but presented inconclusive decisional outcomes. Implementation barriers included lack of design features for connectivity to care, low self-efficacy, and low perceived DA usefulness among healthcare professionals. Other survivor characteristics included age, literacy, preferred timing, and setting.

**Conclusions:**

A diverse range of DAs exists in survivorship care services engagement with favorable knowledge outcomes. Future work should clarify the impact of DAs on decisional outcomes.

**Implications for Cancer Survivors:**

DA characterization and suggestions for prospective developers could enhance support for cancer survivors encountering complex decisions throughout the survivorship continuum.

**Supplementary Information:**

The online version contains supplementary material available at 10.1007/s11764-022-01230-y.

## Introduction

Cancer survivors continually encounter a myriad of health decisions in diagnosis, survivorship, and the end of life [1]. According to the National Cancer Institute, the primary treatment is the first cancer treatment that is typically a combination of surgery, chemotherapy, and radiation [2]. As survivors transit into the post-primary treatment phase, the focus shifts from providing treatment to maintaining health and maximizing the quality of life. Thus, survivors face decisions in engaging a diverse range of survivorship care services, addressing surveillance, physical symptoms, psychosocial issues, and preventive health [3–7]. The participation of survivors in such decisions is increasingly being advocated as the perceived involvement in decision-making about follow-up care is associated with better quality of life up to 10 years post-diagnosis [8]. Contrary to treatment-related decisions, decision-making over service engagement involves a broader range of health disciplines and care settings, survivors’ self-efficacy to implement the chosen option, and self-management to sustain behavioral changes [9, 10]. Since informational needs vary by survivorship phase, suboptimal available information, associated health risks, and long-term adverse treatment pose challenges to decision-making [1, 11–13]. Therefore, interventions supporting decision-making should be tailored to the survivorship phases.

Clinicians could assist survivors in making health decisions post-treatment through shared decision-making (SDM), a process by which physicians inform survivors regarding the potential healthcare interventions [14–16]. Decision aids (DAs) are supporting tools that provide evidence-based available care options and their outcomes while incorporating value clarification components to guide users about the care options based on their preferences [17]. In the post-treatment phase, the potential benefit of DAs for decision-making is two-fold. First, systematic reviews have shown that DAs for cancer-related decisions increase knowledge, reduce decisional conflicts, and enhance satisfaction [18–20]. DAs could address the informational needs unique to the survivorship phase post-primary treatment. In addition, DAs could better align decisions and personalize survivorship care to maximize value to each survivor [21]. DAs are empowering tools to improve survivors’ self-management [22, 23] during survivorship with typically reduced clinical touchpoints from the active treatment phase.

Despite the promising utility of DAs in oncology, existing systematic reviews have disproportionately focused on decisions during the active treatment phase [18–20, 24, 25]. The available DAs and evidence that support their application in the post-treatment phase are still unclear. To address this knowledge gap, this systematic review elucidates the existing DAs developed to support cancer survivors’ decisions to engage in cancer survivorship care services after primary treatment until the end of life. Secondary objectives are to assess the acceptability and impact of the DAs and to outline the implementation challenges. The results would reveal the survivorship care areas where DA development efforts could synthesize available evidence to support the DA usage and implementation strategies in promoting routine care integration.

## Methods

This systematic review was performed following a protocol drafted by the research team (unpublished) and was reported according to the Preferred Reporting Items for Systematic Reviews and Meta-Analyses checklist (Supplementary File 1) [26].

### Eligibility criteria

Inclusion criteria were as follows: (1) targeted patients of any age diagnosed with cancer and/or family caregivers involved in the decision of patients’ care; (2) described the development or evaluation of a DA for engaging with survivorship care services for the post-primary treatment phase; and (3) set in any country or setting. No age limits were set to achieve comprehensive search results. Family caregivers that play active roles in decision-making included parents of pediatric, adolescents, and young adults (AYAs), partners, or adult children [27, 28]. Survivorship care services were defined as health services that addressed any domain of the Quality of Cancer Survivorship Care Framework [29]. Survivorship care services are characterized by survivors’ treatment (prevention and surveillance of new cancers and management of physical and psychosocial effects) and general healthcare (surveillance and management of chronic medical conditions, health promotion, and disease prevention). Survivorship care services also feature the contextual domains of healthcare delivery (clinical structure, communication/decision-making, care coordination, patient/caregiver experience). The systematic categorization of services would reveal gaps or opportunities in clinical care, using consistent terminology in cancer survivorship research. Study protocols were included; however, commentaries, author replies, editorials, and expert opinions were excluded. Studies describing DAs for clinical trial participation were excluded as these decisions did not involve the delivery of established routine care services. Non-English studies were excluded from this review.

### Search strategy

Four electronic databases (PubMed, Embase, PsycINFO, and CINAHL) were searched systematically, and studies that fulfilled the eligibility criteria from inception to 29 September 2021 were selected. Additionally, we searched Google Scholar and reviewed reference lists of all included studies. The following keywords were used for the search: cancer survivor, decision aid, and post-treatment phase. The complete search strategy can be found in Supplementary File 2. Two authors (Y.K. and H.Z.) independently assessed the eligibility of each study by reviewing the titles, abstracts, and entire texts. All discrepancies were discussed and resolved by the two authors.

### Data extraction and result synthesis

One author (H.Z.) extracted data from all included studies using a standardized data collection form, and a second author (Y.K.) performed data accuracy checks. The following data were collected: bibliometrics, study characteristics (objective, design, and setting), study participant characteristics, DA characteristics (title, target user, decision of interest, administration format, development framework/methodology, summary of content, and value clarification exercise), key findings of evaluated measures, implementation challenges (anticipated and encountered), and study limitations. Value clarification methods were described using Witteman et al.’s taxonomy [30]. Implementation challenges were mapped to construct the Consolidated Framework for Implementation Research [31]. Evaluation measures were categorized into acceptability, knowledge, and decision-related outcomes according to the Ottawa Patient Decision Aids Research Group [32].

Unique DAs were identified and characterized to fulfill the primary objective. To fulfill the secondary objectives, evaluation outcomes were summarized using descriptive statistics, and measures of association and their associated uncertainty were reported. The qualitative themes and implementation challenges are summarized.

### Study quality assessment

Quality assessments for the various study designs were performed using the following appraisal tools: the Revised Cochrane risk-of-bias tool for randomized controlled trials (RCTs) [33], the Risk Of Bias in Non-randomized Studies of Interventions for non-randomized studies [34], the Joanna Briggs Institute checklist for cross-sectional studies [35], the Critical Appraisal Skills Programme (CASP) qualitative checklist for qualitative studies [36], and Mixed Methods Appraisal Tool for mixed-method studies [37]. Study assessments were performed independently by two authors (Y.K. and H.Z.), and discrepancies were resolved through discussions until a consensus was reached.

## Results

**Fig. 1:**
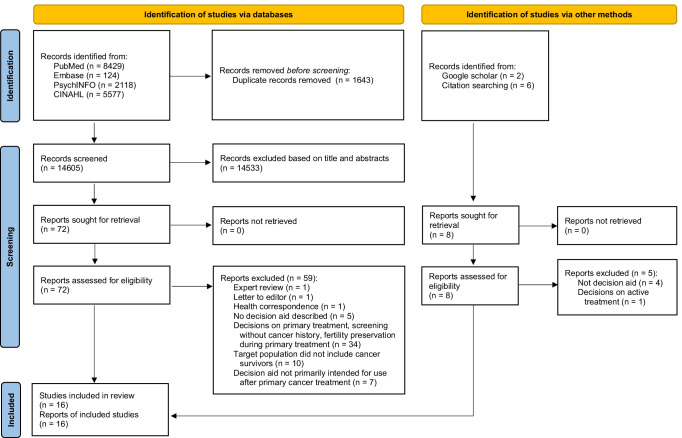
PRISMA 2020 flow diagram for systematic reviews

A total of 16,248 studies were identified on the databases (Fig. 1). After removing 1643 duplicates, the titles and abstracts of 14,605 studies were screened. Full texts were retrieved from 72 studies, and Google Scholar and citation searches identified 8 additional studies. A total of 16 studies satisfied the eligibility criteria and were included in this review [38–53].

### Study characteristics

Selected studies were five RCTs [43, 48, 51–53], two non-randomized trials [46, 49], two cross-sectional studies [42, 47], three DA development studies with qualitative components [41, 44, 45], and two mixed-method studies [40, 50]. A study protocol [39] and a DA descriptive study without evaluation [38] were included. Included studies were conducted in the USA (*n* = 10), Netherlands (*n* = 3), UK (*n* = 1), Germany (*n* = 1), and South Korea (*n* = 1).

### Study quality

All five RCTs were characterized by a high risk of bias [48, 51–53] except one RCT [43]. The two non-randomized trials assessed were at low-risk bias [46] and high-risk bias [49], respectively. Loss-to-follow-up and measurement of subjective outcomes by unblinded investigators led to a high risk of bias. The methodological quality of one cross-sectional study was unclear due to poor reporting of eligibility criteria, study period, and statistical plan [42]. One cross-sectional study was of good quality, but the unclear validity and reliability of its outcome measure might have led to a misclassification bias [47]. All three qualitative studies addressed 9/10 items on the CASP checklist but did not include reflexivity statements [41, 44, 45]. Of mixed-methods studies, one had high methodological quality [50]; the other received a poor rating because they failed to justify the value of mixed-methods and presented poor integration of the quantitative and qualitative components [40]. Detailed assessments are summarized in Supplementary File 3.

### Range of DAs on survivorship services-related decisions

Table 1 summarizes the key characteristics of the 13 unique DAs described in the included studies. A total of eight decisions on survivorship care service engagement were described and mapped to the Quality of Cancer Survivorship Care Framework domains (Table 2). For cancer surveillance, two DAs addressed the prevention of new and/or recurrent breast and ovarian cancer among survivors with breast cancer gene (BRCA) mutations through risk-reduction strategies [41, 44]. One DA focused on lung cancer screening using computerized tomography for Hodgkin lymphoma survivors [40]. To manage late and long-term treatment side effects, a DA intervention on family-building options for women of reproductive age addressed the physical and psychosocial impact on fertility [38]. Another generic DA on insurance plan selection addressed financial toxicity [49]. For health promotion and disease prevention, DAs addressed substance use behavior among AYAs [43] and cardiovascular disease risk reduction among high-risk survivors defined by age and primary treatment history [50]. Lastly, services relating to healthcare delivery include advanced care planning (ACP) discussion, documentation [42, 48, 51–53], and the follow-up intensity of post-treatment consultations [46].

**Table 1:** Range and characteristics of decision aids developed and/or evaluated by included studies

Study, country	Decision aid title	Target user and phase	Decision to be made	Development methodology	Administration	Content	Value clarification exercise
Benedict et al. (2019) [39], Benedict et al. (2021) [38] **Country**: USA	Roadmap to Parenthood	**User**: female cancer survivors aged 15–45 years at risks of gonadotoxic effects **Phase**: post-primary treatment	Parenthood options: natural conception, in vitro fertilization/ surrogacy, and adoption/fostering	**Theory**: self-regulation theory **Guidelines**: (1) IPDAS, (2) Ottawa Decision Support Framework, (3) Guidelines from the Office of Disease Prevention and Health Promotion, NIH, and the Centers for Medicare and Medicaid	Self-administered through a digital website	1) **Context**: information on fertility and cancer treatment effects 2) **Options**: information on family-building options, presented with peer stories depicting each option (personas and storyboards) 3) **Self-assessment**: decision-making readiness, unmet decision support needs self-assessment 4) **Suggestions for next steps**: questions to ask cancer provider and reproductive specialist, financial planning, communication tips with partner, support sourcing 5) Additional resources	Listing concerns—consider pre-identified factors (religious, cultural, and ethical beliefs) when reviewing options and answering open-ended questions
Broadbent et al. (2021) [40] **Country**: UK	Screening to find the early signs of lung cancer after treatment for Hodgkin lymphoma: Helping you decide	**User**: Hodgkin lymphoma survivors **Phase**: post-primary treatment	Lung cancer screening using low-dose CT scans	**Guidelines**: (1) IPDASi, (2) Ottawa Decision Support Framework, (3) “Decisional Needs in Populations” workbook	Self-administered through a booklet in paper format	1) **Context**: information on risk factors for lung cancer following Hodgkin lymphoma treatment, presented in text, icons, and absolute risks 2) **Options**: information on low-dose CT scan procedures presented as images with associated risks and benefits, and screening results interpretation guides 3) **Additional information**: common symptoms of lung cancer 4) **Self-assessment**: textboxes for value clarification exercise and questions listing	Listing pros and cons
Culver et al. (2011) [41] **Country**: USA	What are my options for Breast & Ovarian Cancer Screening and Risk Reduction?	**User**: breast cancer survivors with BRCA mutation **Phase**: newly diagnosed and post-primary treatment	Risk-reducing options: risk-reducing mastectomy, risk-reducing salpingo-oophorectomy, and tamoxifen	**Guidelines**: (1) IPDAS, (2) Ottawa Decision Support Framework	Self-administered through a digital website	1) **Context**: information on risks for a new breast and ovarian cancer 2) **Options**: information on options for breast cancer screening and risk-reduction options, presented using iconic graphs illustrating the relative risk reduction and potential side effects of each option 3) **Self-assessment**: value clarification exercise 4) **Suggestions for next steps**: take-home summary of responses to the value clarification exercise and outlines the next steps to decision-making	Weighing pros and cons—users will rate the importance of each potential benefit and harm associated with the risk-reduction options
Green et al. (2009) [42] **Country**: USA	Making Your Wishes Known: Planning Your Medical Future	**User**: critically ill patients, including cancer as a subgroup **Phase**: any time after diagnosis	Advance care directive creation as part of advance care planning	**Guidelines**: Making Health Communication Programs Work – A Planner’s Guide	Interactive computer program (multimedia with audio, video, and interactivity)	1) **Context**: information on goals, importance, and key components of advance care planning 2) **Options**: information on spokesperson selection, creation of advance care directive, presented using video clips of patient stories and testimonials 3) **Self-assessment**: value clarification exercise, medical wishes 4) **Suggestions for next steps**: summary of values, recommendations on how to initiate and sustain conversations with family members and healthcare professionals	Rating – uses multi-attribute utility theory to account for individual attributes of a decision and rank the relative importance of each attribute
Hollen et al. (2013) [43] **Country**: USA	DecisionKEYS for Balancing Choices: Adolescent Substance Use	**User**: adolescent cancer survivors of childhood cancer **Phase**: disease-free for $\ge$ 5 years and without treatment in the past 2 years	Engaging in substance use behaviors	Not reported	Self-administered through a CD-ROM with videos and interactivity, complemented with counselling session by nurse practitioner for identified high riskers	1) **Preparation**: a decision-making module teaching an easy-recall method for making lifestyle and health-related decisions, presented using a live-action video 2) **Context**: information on the effects of smoking, alcohol, and drug use, presented using a live-action video with role playing of refusal strategies 3) **Options**: interactive module allowing users to make choices under difficult situations associated with substance use and presenting the eventual outcomes based on prior choices 4) **Self-assessment**: value clarification exercise	Listing pros and cons—uses a tailored decisional balance sheet
Kautz-Freimuth et al. (2021) [44] **Country**: Germany	Untitled	**User**: female breast cancer survivors with pathogenic BRCA mutations **Phase**: post-primary treatment	Risk-reducing options: intensified breast cancer screening and aftercare program, risk‐reducing contralateral mastectomy, and risk-reducing salpingo-oophorectomy	**Guidelines**: (1) IPDAS, (2) Ottawa Decision Support Framework	Self-administered through printed paper brochures (electronic versions that can be downloaded in PDF format were eventually available)	1) **Context**: information on BRCA mutations, risks for a new breast and ovarian cancer 2) **Options**: information on options for cancer screening and risk-reduction options, presented using overview tables with pros and cons 3) **Self-assessment**: value clarification exercise 4) **Suggestions for next steps**: preparation for doctor consultation 5) Additional resources: tips, helpful contacts, glossary	Viewing pros and cons and reviewing a list of values and preferences pre-identified
Klaassen et al. (2018) [45, 46], Klaassen et al. (2020) [47] **Country**: Netherlands	Untitled	**User**: breast cancer survivors **Phase**: post-primary treatment	Aftercare options: consultations in hospital (intensive) or scheduled telephonic consultations and/or consultations in hospital on demand (less intensive)	**Guidelines**: IPDASi	Administered during consultation with a health professional through a digital website	1) **Context**: information on late side effects of primary treatment, follow-up frequency based on national guidelines 2) **Self-assessment**: value clarification exercise 3) **Options**: information on the intensity of follow-up options at hospitals and intuitive preference assessment 4) **Suggestions for next steps**: overview of (mis)match between aftercare options and individual preferences via an opinion grid to be discussed with healthcare professionals	Rating – users will indicate the importance of each aftercare characteristic (derived from preliminary studies) and rank the 5 most important characteristics
Matlock et al. (2014) [48] **Country**: USA	Looking Ahead: Choices for medical care when you're seriously ill	**User**: critically ill patients and their decision-makers, including cancer as a subgroup **Phase**: any time after diagnosis	Option of palliative care and advance care planning	**Guidelines**: none specified General methodology included (1) literature review, (2) focus groups with hospice, palliative care providers, and caregivers, (3) expert review	Self-administered through a booklet and DVD at users' convenience before, during, or after the palliative care team consultation	1) **Context**: information on goals, importance, and key components of advance care planning 2) **Options**: information on palliative care, advance care planning 3) **Self-assessment**: value clarification exercise	Present, but details not reported
Politi et al. (2020) [49] **Country**: USA	Improving Cancer Patients’ Insurance Choices (I Can PIC)	**User**: cancer survivors **Phase**: $\le$ 5 years from cancer diagnosis	Health insurance plans	**Guidelines**: none specified General methodology included (1) qualitative interviews with cancer survivors that explored the challenges they faced selecting and using health insurance, (2) personalized estimations of annual healthcare expenses based on the Medical Expenditure Panel Survey, (3) plans selected by study team after review based on importance	Self-administered through a digital website	1) **Context**: information on health insurance using plain language, graphics, and examples from survivors 2) **Options**: provide personalized cost estimates of annual healthcare expenses across plan types available to user 3) **Additional information**: list of financial and emotional support resources	Not reported
Raghunathan et al. (2020) [50] **Country**: USA	Modified statin risk communication tool modeled after the validated Statin Choice Decision Aid	**User**: high risk cancer survivors (e.g., aged < 40 years at time of cancer diagnosis; received $\ge$ 2,000 cGy to the heart/chest; received radiation $\ge$ 10 years prior) **Phase**: post-primary treatment	Usage of statin therapy to reduce cardiovascular disease risk	**Guidelines**: none specified General methodology included iterative engagement with care providers, patient education specialists, and graphic designers	Administered by trained physicians and nurse practitioners during consults (DA format is unclear)	1) **Context**: a pictorial representation of absolute coronary heart disease risk in survivors of childhood, adolescent, and young adult cancer treated with radiation to the chest 2) **Options**: information on statin, cost, and side effects, associated cardiovascular disease absolute risk reduction	Not reported
Smith et al. (2020) [51] **Country**: USA	Four Conversations	**User**: patients with metastatic breast cancer **Phase**: any time after diagnosis	Advance care directive creation	Not reported	Administered through a digital website, part of a 4-week program with a specially trained ‘‘Pillar Guide’’ over e-mail and/or telephone to discuss learning points	1) **Context**: information on end-of-life reflections and medical wishes 2) Initiation of a series of conversations including informative conversation with healthcare providers, supportive conversation with family and friends, inspirational conversation about spirit, honest conversation with self	Not reported
Vogel et al. (2013) [52] **Country**: USA	Together	**User**: women with stage III/IV or recurrent ovarian cancer **Phase**: post-primary treatment and any time after recurrent cancer diagnosis	Advance care directive creation and participation in Palliative Care consultation as part of advance care planning	**Guidelines**: none specified General methodology included a day-long “design event”, during which clinical, information and decision experts and ovarian cancer patients and their families discussed ideas for improved cancer care	Self-administered through a digital website	1) **Context**: information on ovarian cancer, palliative care, emotional well-being, social support, practical considerations, physical well-being and communication 2) **Options**: health directive form and goal-setting options 3) **Self-assessment**: distress monitoring using the distress thermometer, value clarification exercise, journal 4) **Suggestions for next steps**: preparation for doctor consultation 5) Complementary website for caregivers: similar information to the patient version with a separate discussion forum, and an option to view a summary page of the patient’s questions and distress levels	Rating and listing pros and cons with weighing – uses interactive PDF of the Ottawa Personal Decision Guide to help participants weigh risks and benefits associated with the decision based on how much each factor matters to the users
Yun et al. (2019) [53] **Country**: South Korea	Advance care planning	**User**: patients with advanced cancer **Phase**: any time after diagnosis	Advance care directive creation as part of advance care planning	**Guidelines**: (1) IPDAS, (2) Smart Management Strategy for Health Assessment Tool	Administered through a video and a companion book	1) **Context**: understanding the need for participation in decision-making, the necessity to prepare for death, perceptions of death 2) **Options**: advance care planning and preparation 3) Additional information: requirement, methods, and barriers of communication with medical staff, friends, and family	Not reported

Abbreviations: *BRCA*, breast cancer gene; *CD-ROM*, compact disc read-only memory; *cGy*, centigray; *CT*, computerized tomography; *DVD*, digital video disc; *IPDAS*, International Patient Decision Aid Standards; *IPDASi*, International Patient Decision Aid Standards Instrument; *NIH*, National Institutes of Health

**Table 2:** Survivorship care domains addressed by included decision aids

Study, country	Domains pertaining to cancer and its treatment			Domains pertaining to general healthcare	Contextual domains of healthcare delivery	
	Prevention and surveillance for recurrence and new cancers	Surveillance and management of physical effects	Surveillance and management of psychosocial effects	Health promotion and disease prevention	Clinical structure	Communication/decision-making
Benedict et al. (2019) [39], Benedict et al. (2021) [38] **Country**: USA		X Family building	X Family building			
Broadbent et al. (2021) [40] **Country**: UK	X Lung cancer screening					
Culver et al. (2011) [41] **Country**: USA	X New/ recurrent breast and ovarian cancer risk-reduction strategies					
Green et al. (2009) [42] **Country**: USA						X Advance care planning discussion and/or documentation
Hollen et al. (2013) [43] **Country**: USA				X Regulating substance use behavior		
Kautz-Freimuth et al. (2021) [44] **Country**: Germany	X New/ recurrent breast and ovarian cancer risk-reduction strategies					
Klaassen et al. (2018) [45, 46], Klaassen et al. (2020) [47] **Country**: Netherlands					X Aftercare follow-up intensity	
Matlock et al. (2014) [48] **Country**: USA						X Advance care planning discussion and/or documentation
Politi et al. (2020) [49] **Country**: USA			X Insurance plans to prevent financial toxicity			
Raghunathan et al. (2020) [50] **Country**: USA				X Cardiovascular disease risk reduction		
Smith et al. (2020) [51] **Country**: USA						X Advance care planning discussion and/or documentation
Vogel et al. (2013) [52] **Country**: USA						X Advance care planning discussion and/or documentation
Yun et al. (2019) [53] **Country**: South Korea						X Advance care planning discussion and/or documentation

Most DAs were specific to the post-primary treatment survivorship phase, except ACP and insurance plan decisions after diagnosis [42, 48, 49, 51–53]. The users of the two DAs of ACP were broad, including the cancer population as a subgroup [42, 48]. The remaining DAs had different specificity based on (1) cancer type such as breast, ovarian, Hodgkin lymphoma, (2) cancer mutation status, (3) cancer staging, (4) primary treatment received, and (5) the AYA age group.

### Design features of DAs

Approximately half (7/13) of the DAs specified the guidelines for DA development, with the International Patient Decision Aid Standards and Ottawa Decision Support Framework most cited [38, 40, 41, 44, 46, 53]. The remaining DAs were developed through a methodology involving a literature review of available evidence and qualitative discussions with target users. The majority (8/13) of the DAs employed digital websites or computer programs for the DAs format [39, 41–43, 46, 49, 51, 52]. Two DAs employed the paper format [40, 44], and two DAs adopted a hybrid format [48, 53]. Most DAs were for self-administration at users’ convenience, and three DAs were administered during consultations with healthcare professionals [46, 50, 51].

### Contents features of DAs

All DAs generally followed a similar structure for content presentation. The context of the decision was first described, explaining the heightened risk of recurrent or second cancer, late or long-term physical and psychosocial effects, and the importance of goal setting near end-of-life. Next, a list of possible options was presented using methods such as storyboards or survivors’ anecdotes [38, 49], actual imaging results for the case of low-dose CT scan [40], pictorial representation of risks [41, 50], and comparison tables [44]. Additionally, the DA on insurance plan selection provided personalized cost estimates for each option [49]. Nine DAs used value clarification exercises to help survivors understand their preferences before guiding them about the application of the DA results for consultation with care providers [38, 40, 41, 43, 44, 46]. The methods employed in the exercises included rating the relative importance of different attributes in decision-making [42, 46, 52], listing pros and cons of available options with (*n* = 2) [41, 52] and without weighing (*n* = 2) [40, 43], listing concerns (*n* = 1) [38], and viewing a pre-identified list of values, options’ pros and cons (*n* = 1) [44]. The method was unclear in one DA [48]. The DA addressing follow-up intensity further employed an option grid to visually compare the congruency between the user’s preference and intuitive choice [46]. The DA on substance use among AYA had additional unique content features [43]. Before introducing the decision context, users were to complete a general health decision-making module. Furthermore, the options were not presented as organized information. Instead, users were guided to explore the consequences of each chosen option via dynamic pathways in an interactive program.

### Acceptability of DAs

Seven studies evaluated the acceptability of developed DAs [40, 42, 43, 48, 50–52] (Table 3). Information clarity was rated favorably for the DA on substance use [43], and 58% of participants found the information clarity of the DA on statin use highly acceptable [50]. The DA on lung cancer screening was rated favorably for balance (76.3%) and the right amount of information (94.7%) [40]. DAs targeting ACP received high satisfaction scores [42], and $\ge$ 88% of participants would recommend the DA to others facing similar decisions [48, 51, 52]. In one study which compared DA usage to an active comparator using an informational website, no significant differences were found in the satisfaction of the amount (*P* = 0.054) and quality (*P* = 0.119) of information between the groups [52].

**Table 3:** Summary of studies reporting the development or evaluation of decision aids

Study, country, DA	Objective/study design	Participants	Outcomes	Implementation challenges	Limitations
Broadbent et al. (2021) [40] **Country**: UK **DA**: Screening to find the early signs of lung cancer after treatment for Hodgkin lymphoma: Helping you decide	**Objective**: develop and preliminary test a lung cancer screening DA among survivors of Hodgkin lymphoma and practitioner stakeholders **Design**: mixed methods (DA development study comprising qualitative focus groups and interviews, and cross-sectional study)	**Setting**: community **Cancer type**: Hodgkin lymphoma **Age** [median (range)]: survey [44 (21–71)], focus group 1 (26–60), focus group 2 (21–71) **Gender** (female): survey (79%), focus group 1 (50%), focus group 2 (100%) **N**: survey (38), focus groups (11), lymphoma practitioners (11)	Acceptability •86.6% found the length and 94.7% found the amount of information to be just right •76.3% found the DA to be balanced •All participants said they would find the DA useful Knowledge •Improved lung cancer risk and screening-related knowledge after DA usage (median correct responses: pre-DA = 68% vs. post-DA = 93%, *P* < 0.001) •60.6% said they would not seek out more information Decisional outcomes •Reduced decisional conflict after DA usage [median (IQR) score: pre-DA = 67.5 (40) vs. post-DA = 0 (10), *P* < 0.001] Choice predisposition •DA did not significantly change intention to participate in future lung cancer screening program (*P* = 0.21) Qualitative themes •Explored DA usage, factors influencing lung cancer screening participation decisions, and information provision and support	Not reported	•Limited generalizability to individuals with low literacy levels, current smokers, and non-white ethnicities •Representativeness of intended target group may be compromised as some participants may not be at excess risk for lung cancer
Culver et al. (2011) [41] **Country**: USA **DA**: What are my options for Breast & Ovarian Cancer Screening and Risk Reduction?	**Objective**: (i) understand the unique decision-making needs of women with breast cancer and BRCA mutations, and (ii) develop an effective, easily accessible and updateable DA for this high-risk population **Design**: DA development study (comprising qualitative focus groups and interviews)	**Setting**: outpatient Survivors **Cancer type**: breast **Age** [mean (range)]: phase 1 focus groups [50.8 (32–67)], phase 2 focus groups [51.7 (42–64)] **Gender** (female): 100% **N**: phase 1 focus groups (11), phase 2 focus groups (9) Others **N**: genetics (15) and oncology (11) professionals, advocates (12)	Qualitative themes from phase 1 focus groups (survivors only) •Explored decision-making factors and process regarding risk reducing mastectomy and oophorectomy •Elicited benefits and harms to be used for value clarification exercise in actual DA Qualitative themes from phase 1 focus groups (mixed) •Participants generally agreed that the DA would facilitate processing of information •Positive feedback on clarification of values ranking exercise as it allows for a less emotionally intense process and removes external pressures by allowing survivors to complete the exercise independently •Most participants preferred graphs and numbers to convey risk reduction instead of facial icons; and found the use of ribbons to illustrate the level of evidence to be confusing •Most participants wanted more information regarding the benefits, limitations, and risks of various options •Participants suggested variables that could be used to tailor and personalize the DA	DA characteristics A web-based format required computer access but had advantages of rapid update, ease of dissemination, ability to track compliance and usage patterns Individual survivor characteristics •State of change: there was lack of consensus over the appropriate timing to introduce DA relative to receiving genetic risk result and required assessment of survivors’ emotions and readiness •Setting preference: survivors preferred using DA in the clinical setting, allowing a closer connection to the medical team	•Generalizability of viewpoints to BRCA mutation carriers residing outside Southern California may be limited •Selection bias: participants were all willing to evaluate a computerized version of the DA
Green et al. (2009) [42] **Country**: USA **DA**: Making Your Wishes Known: Planning Your Medical Future	**Objective**: describe the development of an innovative, multimedia DA for advance care planning designed to overcome common problems with standard advance directives **Design**: cross-sectional pilot study	**Setting**: outpatient **Cancer type**: mixed (pilot 2) **Age** (mean): pilot 1 (52), pilot 2 (57) **Gender** (female): pilot 1 (68%), pilot 2 (71%) **N**: pilot 1 (50), pilot 2 (34)	Acceptability (pilot 1: participants without cancer*)* •An average of 106 min was needed to complete the DA, and participants indicated that this duration of time was not burdensome •Overall mean satisfaction score = 8.5 (range: 1 = not at all satisfied, 10 = extremely satisfied) •Mean satisfaction score of how DA improved knowledge and helped in decision-making = 4.2 (range: 1 = very dissatisfied, 5 = very satisfied) Acceptability (pilot 2: participants with cancer*)* •Overall mean satisfaction score = 8.5 (range: 1 = not at all satisfied, 10 = extremely satisfied) Other outcomes (pilot 2: participants with cancer*)* •High accuracy of computer-generated advanced directive in representing users’ wishes [mean score (1 = not at all accurate, 7 = very accurate): pre-edit = 5.5 vs. post-edit = 6.5, *P* < 0.001] •No changes to hopefulness, hopelessness, or anxiety after DA usage	*Anticipated challenges*: Individual survivor characteristics •Clinical and sociodemographic: a computer program format may be too complex for those who are old, sick, or poorly educated •Competence: participants may cite inability to forecast future wishes/preferences meaningfully and reliably	Not reported
Hollen et al. (2013) [43] **Country**: USA **DA**: DecisionKEYS for Balancing Choices: Adolescent Substance Use	**Objective**: test a DA for cancer-surviving adolescents aimed at difficult decisions related to engaging in substance use risk behaviors for immediate and sustained changes **Design**: randomized controlled trial *Intervention*: DA *Control*: usual care	**Setting**: outpatient **Cancer type**: mixed **Age** [mean (SD)]: 16.3 (1.6) **Gender** (female): 53% **N**: intervention (102), control (111)	Acceptability (*intervention group*) •95% perceived the DA to be helpful •94% found the description of possible late effects of cancer treatment to be clear •98% found the substance use risk behavior fact sheet and the information on risk behaviors to avoid to be clear •98% believed in the usefulness of decision-making theory and were able to take steps to make an important decision Decisional outcomes •No significant differences in quality of decision-making were found between groups when assessed at 6-month (*P* = 0.77) and 12-month timepoint (*P* = 0.72) Choice predisposition •Transient significant increased attitude towards substance use behavior was found at the 6-month timepoint with DA usage [score difference (SE): 5.75 (2.84), *P* = 0.04], but the effect was not sustained at 12-month timepoint [score difference (SE): − 1.56 (2.66), *P* = 0.56] •No changes in substance (cigarettes, alcohol, illicit drugs) use behaviors after DA usage (quantitative measures not reported)	*Anticipated challenges*: Individual survivor characteristics •Intellectual ability: it may be harder to implement DA among individuals with cognitive impairment •Social environment: it may be harder to use DA if survivors’ family and close friends modeled substance use •DA usage may interfere with school commitments in the adolescent age group	•Limited generalizability to other social changes in attitudes towards alcohol use, especially when 90% of participants’ household members modeled substance use of some type •Internal validity may have been affected by a higher-than-expected variability or the potential for smaller effect size •The Decision-Making Quality Scale may not be sensitive enough as a baseline measure of decision-making or minimally important difference needs to be re-examined
Kautz-Freimuth et al. (2021) [44] **Country**: Germany	**Objective**: develop a DA to support the decision-making process for women carrying BRCA mutations with and without breast cancer **Design**: DA development study (comprising qualitative focus groups and interviews)	**Setting**: outpatient **Cancer type**: breast **Age**: not reported **Gender** (female): 100% **N**: focus groups for DA design (10), user testing (5)	Qualitative themes for DA design •Positive feedback on volume and detail of information •More information was requested for procedures after each risk-reduction option, cancer recurrence, and biological parameters •Integrated value clarification exercise was perceived positively, but suggestions were made to replace pre-identified list of values with a blank space to allow users to formulate and clarify their own values Qualitative themes from user testing •Participants rated DA positively, particularly in length, balanced presentation, usefulness for decision-making, sufficient information to make decisions, satisfaction, and likelihood of recommendation to others	Not reported	•Lack of a systematic evidence review for medical content in the DA •Selection bias: capture viewpoints from a purposively selected volunteer target group
Klaassen et al. (2018) [45] **Country**: Netherlands **DA**: Untitled—aftercare options	**Objective**: investigate and compare patients’ and health professionals’ experiences and preferred decision-making processes, preferences for availability of options in aftercare and the DA format **Design**: qualitative	**Setting**: outpatient **Cancer type**: breast **Age** [mean (range)]: 62 (49–75) **Gender** (female): 100% **N**: survivors (11) Others **N**: health professionals (8)	Qualitative themes •Survivors reported a lack of opportunity to discuss available aftercare options •Health professionals perceived their role in the decision-making process as evidence-based medical information provider •Survivors indicated that their decisions were based more on intuitive processes •Both survivors and health professionals reported that a DA would be a helpful tool	DA characteristics Disagreement in DA format between survivors and health professionals who preferred paper- and digital-based format, respectively Individual survivor characteristics •State of change: there was lack of consensus over the appropriate timing to introduce DA during or after primary treatment between survivors and health professionals •Setting preference: both survivors and health professionals agreed with using the DA in the clinical setting	Limited generalizability beyond the southeast region of the Netherlands
Klaassen et al. (2018) [46] **Country**: Netherlands **DA**: Untitled—aftercare options	**Objective**: test the effects of a DA on patient-perceived shared decision-making, patient decision evaluation, aftercare choice, and hospital costs **Design**: prospective before-and-after *Intervention*: DA + aftercare decision consultation *Control*: aftercare decision consultation	**Setting**: hospitals **Cancer type**: breast **Age** [mean (SD)]: intervention [58.4 (11.0)], control [59.7 (8.6)] **Gender** (female): 100% **N**: intervention (43), control (44)	Decisional outcomes •No significant difference in perceived shared decision-making was found between groups (*P* = 0.31) •No significant differences in decision satisfaction and uncertainty were found between groups, immediately (P = 0.27) and at 3 months (*P* = 0.40) after aftercare consultations •No significant differences in perceived informed choice were found between groups, immediately (*P* = 0.83) and at 3 months (*P* = 0.11) after aftercare consultations •DA usage was associated with a higher perceived control over choice 3 months after aftercare consultations [mean (SD) score (range: 1–5): intervention = 2.37 (1.16) vs. control = 1.75 (0.64), *P* = 0.01] Choice predisposition •A greater proportion of participants chose less intensive aftercare after DA usage (intervention = 51% vs. control = 29%, *P* = 0.04) Other outcomes •Consultation time (minutes) increased with DA usage [mean (SD): intervention = 42.3 vs. control = 29.8, *P* < 0.01] •No significant difference in hospital costs (€) was found between groups 3 months after aftercare consultations (*P* = 0.38)	Not reported	Short follow-up period of 3 months may not allow inferences about preference stability and hospital cost since traditional aftercare consisted of consultations once every 3 months
Klaassen et al. (2020) [47] **Country**: Netherlands **DA**: Untitled—aftercare options	**Objective**: identify facilitators and barriers for use of breast cancer aftercare DA in healthcare practice **Design**: cross-sectional study	**Setting**: hospitals **Cancer type**: NA Healthcare professionals **Age** [mean (SD)]: intenders to adopt DA [47.3 (7.6)], non-intenders [48.5 (8.0)] **Gender**: not reported **N**: intenders (44), non-intenders (37)	•A higher perceived relevance of the DA for the patient by healthcare professionals was associated with a higher intention to adopt DA (OR = 7.40; 95% CI 1.85–29.58, *P* = 0.01) •Increased healthcare professionals’ self-efficacy was associated with a higher intention to adopt DA (OR = 14.56; 95% CI 1.09–194.09, *P* = 0.04)	Inner setting Learning climate: poor self-efficacy could be mitigated by creating a conducive learning environment where healthcare professionals can learn from one another’s experience with the DA Process Low perceived usefulness of DA could be addressed by adequate feedback on results of DA evaluation	Misclassification bias may arise from assuming neutral responses as non-intenders
Matlock et al. (2014) [48] **Country**: USA **DA**: Looking Ahead: Choices for medical care when you’re seriously ill	**Objective**: assess the feasibility and acceptability of a DA designed to inform and empower patients and family members facing advanced or terminal illness **Design**: pilot randomized controlled trial *Intervention*: DA + palliative care service *Control*: palliative care service	**Setting**: inpatient Survivors **Cancer type**: mixed (subgroup of study population) **Age** [median (range)]: intervention [54 (28–66)], control [55 (31–78)] **Gender** (female): intervention (78%), control (55%) **N**: intervention (9), control (11) Survivors’ decision makers **N**: intervention (16), control (15)	Acceptability (*intervention group*) •Most participants felt the DA contained the right amount of information (76%), was balanced (94%) and 88% would definitely recommend it to other people facing the same decision Knowledge •No significant difference in knowledge on important aspects of decision-making during advanced illness was found between groups (*P* = 0.35) Decision-related outcomes •No significant difference in decisional conflict was found between groups (*P* = 0.41) Qualitative themes •DA increased empowerment and control, validating decisions, heightening motivation for completing one’s own advance directives, and allowed anticipated impact on future discussions with physicians	Individual survivor characteristics State of change: participants preferred the introduction of DA at an earlier point in the disease process	•Suboptimal outcomes choice as exit interviews suggested that outcomes such as empowerment, confidence or self-efficacy may be more appropriate •High attrition rate may have contributed to confounding •Comparator choice of palliative care consultation may be analogous to some form of intervention, biasing results to the null on the eventual observed DA’s impact
Politi et al. (2020) [49] **Country**: USA **DA**: Improving Cancer Patients’ Insurance Choices (I Can PIC)	**Objective**: evaluate a web-based DA designed to support health insurance decisions of patients with cancer and survivors **Design**: randomized controlled trial (with baseline adjustment) *Intervention*: DA *Control*: a health insurance worksheet developed by the ACS CAN	**Setting**: outpatient and community **Cancer type**: mixed **Age** [mean (SD)]: intervention [52.4 (10.0)], control [52.9 (9.4)] **Gender** (female): intervention (60%), control (68%) **N**: intervention (106), control (100)	Knowledge •DA usage improved health insurance knowledge immediately [mean (SD) scores: intervention = 89.29 (12.31) vs. control = 74.38 (17.98), P < 0.0001] and 3–6 months after [mean (SD) scores: intervention = 82.07 (18.00) vs. control = 74.86 (17.32), P = 0.002]. [48, 51–53] •A higher proportion of participants were very confident of understanding health insurance terms immediately after using DA (intervention = 53.77% vs. controls = 32.32%, *P* = 0.002), but no significant difference is observed for estimating cost of care immediately after using DA (*P* = 0.501) Decisional outcomes •No significant difference in decision self-efficacy was found between groups immediately (*P* = 0.522) and 3–6 months (*P* = 0.103) after baseline •No significant difference in decisional conflict was found between groups immediately (*P* = 0.094) and 3–6 months (*P* = 0.766) after baseline Other outcomes •No significant difference in financial toxicity was found between groups (*P* = 0.345) •No significant differences in proportion of participants who delayed or avoided general care (*P* = 0.605) and cancer care (*P* = 0.736) due to cost were found between groups	Individual survivor characteristics •State of change: there was an inertia to change insurance plans without a prompt among survivors •Limited applicable health insurance plans available for choice	•Selection bias: relatively educated sample, majority had health insurance options available and could not switch plans even if they wanted to, resulting in limited plan choices/flexibility •Participants might not have needed to seek care during the short follow-up period •No healthcare system cost captured •Inability to pair study enrollment with variable open enrollment periods provided by employers
Raghunathan et al. (2020) [50] **Country**: USA **DA**: Modified statin risk communication tool modeled after the validated Statin Choice DA	**Objective**: pilot test an adapted statin therapy risk communication tool for use among high-risk cancer survivors to improve shared decision-making regarding cardiovascular disease risk reduction **Design**: mixed methods (pilot non-randomized controlled trial and qualitative interviews) *Intervention*: DA + cardiovascular risk discussion *Control*: cardiovascular risk discussion	**Setting**: outpatient **Cancer type**: mixed **Age** [median (IQR)]: intervention [46.0 (37.5–52.5)], control [44.5 (34.25–55.5)] **Gender** (female): intervention (75%), control (59%) **N**: intervention (24), control (22)	Acceptability (*intervention group*) •92% found the information amount to be highly acceptable •58% found the information clarity to be highly acceptable Knowledge *(full cohort)* •Half of the cohort answered 10-year myocardial infarction risk correctly •72% answered myocardial infarction risk with statin correctly • > 50% responded “do not know” to side effects of statin Decisional outcomes •No significant difference in decisional conflict was found between groups (*P* = 0.231) Qualitative themes •Participants endorsed feeling more clarity through both conversation with the clinician and DA usage •Apprehension in starting medication related to side effects •Participants were comfortable with the eventual decision, regardless of whether therapy changed after the conversation with the clinician using the DA	Not reported	Selection bias: overrepresentation of highly educated population, biasing results of the full cohort towards higher knowledge scores
Smith et al. (2020) [51] **Country**: USA **DA**: Four Conversations	**Objective**: test the efficacy of a personalized, online coping, and DA curriculum for patients with metastatic breast cancer, in terms of completing advance directive, program satisfaction, and impact on decision-making and quality of life **Design**: randomized controlled trial *Intervention*: DA *Control*: wait-listed usual care conditions	**Setting**: outpatient **Cancer type**: breast **Age** [mean (SD)]: intervention [52.7 (10.7)], control [54.3 (11.2)] **Gender** (female): 100% **N**: intervention (110), control (142)	Acceptability (*intervention group*) •90% would recommend the DA to others and learned $\ge$ 1 new thing that helped them feel more peaceful •87% felt that the online format was easy to follow and appropriate for the goals of the program •Cited reasons for lack of perceived helpfulness: (1) logistic programmatic issues; (2) emotional discomfort; and (3) bad fit Decisional outcomes •No significant differences in decisional conflict reduction (*P* = 0.07) and decision self-efficacy (*P* = 0.61) were found between groups at 4 weeks from baseline •62% of the intervention group felt that the DA prepared them “a great deal” or “quite a bit” to make a better decision •85% of the intervention group were confident in their ability to communicate their wishes for treatment and end-of-life to their healthcare clinicians Other outcomes •No significant differences in physical (*P* = 0.14) and mental (*P* = 0.16) quality of life scores were found between groups at 4 weeks from baseline	Not reported	•Limited generalizability to metastatic breast cancer patients with low education levels •High attrition rate may have contributed to confounding •Lack of outcome measures to assess negative aspects (e.g., anxiety provoking elements)
Vogel et al. (2013) [52] **Country**: USA **DA**: Together	**Objective**: develop and pilot-test a web-based tool to promote advance care planning for women with ovarian cancer **Design**: pilot randomized controlled trial *Intervention*: DA *Control*: website with usual care information	**Setting**: outpatient **Cancer type**: epithelial ovarian, primary peritoneal or fallopian tube **Age** [mean (SD)]: intervention [59.6 (10.0)], control [55.5 (8.4)] **Gender** (female): 100% **N**: intervention (20), control (15)	Acceptability •No significant differences in satisfaction of the amount (*P* = 0.054) and quality (*P* = 0.119) of information were found between groups • > 90% of participants in both groups would recommend the respective website to others Decision-related outcomes •No significant difference in decisional conflict was found between groups (*P* = 0.519) Choice predisposition •No significant differences in completion of advance healthcare directive (*P* = 0.220) and decision-making regarding palliative care (*P* = 0.440) were found between groups Other outcomes •More time (minute) was spent on the DA than the usual care [mean (SD): intervention = 19.9 (14.6) vs. control = 15.9 (8.7), *P* = 0.049] •Access to DA and usual care website were comparable during study period (median number of logins: intervention = 4.5 vs. control = 4.5)	DA characteristics •Design: limited user engagement through discussion forums and lacked real-time connectivity to care providers •Complexity: lacked navigation guide on DA usage and lacked prompts and triggers to action for users to follow Individual survivor characteristics State of change: survivors further away from diagnosis perceived the DA to be less useful	•Selection bias: overrepresentation of individuals who are educated, white, insured, and with computer access •Caregivers were not successfully engaged
Yun et al. (2019) [53] **Country**: South Korea **DA**: Advance care planning	**Objective**: test whether a DA explaining advance care planning is more likely than the control to help patients with advanced cancer understand advance care planning and select a preference for their eventual end-of-life care **Design**: randomized controlled trial *Intervention*: DA *Control*: video and workbook that discussed cancer pain control	**Setting**: inpatient and outpatient **Cancer type**: mixed **Age** [mean (SD)]: intervention [58.1 (11.9)], control [57.1 (11.0)] **Gender** (female): intervention (61%), control (62%) **N**: intervention (104), control (100)	Knowledge •DA usage increased knowledge of advance care planning and cardiopulmonary resuscitation when assessed at 7 weeks after baseline [mean (SD) score: intervention = 5.12 (0.97) vs. control = 4.66 (1.11), *P* = 0.005] Decision-related outcomes •At 7 weeks after baseline, no significant difference in decisional conflict was found between groups (*P* = 0.681) Choice predisposition •DA reduced the preference for active and life-prolonging treatment and increased the preference for hospice care as a primary outcome across life expectancy subgroups (1 year, months, weeks) at baseline, but this effect is sustained only for hospice care 7 weeks after baseline •At 7 weeks after baseline, no significant differences in proportion of participants intending to document advance care planning (*P* = 0.007*), having end-of-life discussion (*P* = 0.127), and having documentation of own preference of end-of-life care (*P* = 0.577) were found between groups •At 7 weeks after baseline, no significant difference in proportion of participants who prefer active roles in decision-making was found between groups (*P* = 0.583) Other outcomes •No significant differences in anxiety (*P* = 0.917) and depression (*P* = 0.321) were found between groups 7 weeks after baseline	Not reported	•Limited generalizability to non-Koreans, non-cancer severe illness, and more diverse population •Outcome ascertainment bias from data collection by unblinded research assistants •Change in end-of-life care preferences questionnaire was not validated •Misclassification bias may arise from weak and vague difference between “active” and “life-prolonging treatment” distinguishment

Abbreviations: *BRCA*, breast cancer gene; *CI*, confidence interval; *DA*, decision aid; *IQR*, interquartile range; *OR*, odds ratio; *SD*, standard deviation

^*^P < 0.006 denotes statistical significance after Bonferroni correction

### Impact of DAs (Table 3)

#### Knowledge

Five studies assessed survivors’ knowledge using investigator-designed questionnaires [40, 48–50, 53]. DAs were associated with improved knowledge when assessed immediately (median correct responses for lung cancer screening: pre-DA = 68% vs. post-DA = 93%, *P* < 0.001) [40] and several weeks after [mean (SD) knowledge score of ACP 7 weeks after baseline: intervention = 5.12 (0.97) vs. control = 4.66 (1.11), *P* = 0.005] [53]. Contrarily, a pilot RCT did not report significant improvement in ACP knowledge after usage, possibly limited by the statistical power [48]. A study reported that the improvement in knowledge of health insurance plans immediately after DA usage (*P* < 0.0001) was sustained when reassessed 3–6 months later (*P* = 0.002) [49]. The last study evaluated the knowledge of the intervention and control groups and revealed gaps in knowledge in cardiovascular disease risk and statins side effects [50].

#### Decisional outcomes

The most reported decisional outcome was the decisional conflict that measured survivors’ uncertainty about decision-making. Of seven studies [40, 48–53], only one cross-sectional study reported reduced decisional conflict immediately after using DA for lung cancer screening [median (IQR) score: pre-DA = 67.5 (40) vs. post-DA = 0 (10), *P* < 0.001] [40]. While pilot trials were limited by statistical power, three full-sized RCTs reported no significant differences between controls and DA usage for the insurance plans and ACP [49, 51, 53]. Two RCTs reported no significant impact of DAs on decision self-efficacy—the measure of survivors’ self-confidence to make decisions [49, 51]. Other decisional outcomes evaluated, not showing significant improvement with DA usage, were the quality of decision-making [43] and perceived shared decision-making [46]. The DA on breast cancer aftercare follow-up intensity showed that DA usage was associated with a higher perceived control over choice 3 months after aftercare consultations [mean (SD) score (range: 1–5): intervention = 2.37 (1.16) vs. control = 1.75 (0.64), *P* = 0.01] [46].

#### Choice predisposition

Five studies evaluated the impact of DAs on survivors’ propensity to make a choice [40, 43, 46, 52, 53]. Compared with controls, two studies reported no significant differences in the intention to complete advanced healthcare directives [52, 53]. The DAs did not significantly change the intention of survivors to participate in future lung screening programs [40]. Moreover, transient improvement in attitude toward substance use after the DA did not translate to a change in substance use behavior [43]. Only the study on DA for breast cancer aftercare follow-up intensity reported a higher proportion of survivors in the intervention group choosing a less intensive follow-up modality (intervention = 51% vs. control = 29%, *P* = 0.04) [46].

#### Other outcomes

DA usage increased consultation time (minutes) [mean (SD): intervention = 42.3 vs. control = 29.8, *P* < 0.01] but was not associated with hospital cost difference over a 3-month follow-up period [46]. Psychological constructs such as hopefulness, hopelessness, anxiety, and depression did not differ from the controls following DA for ACP [42, 53]. Additionally, physical and mental quality of life scores did not differ from controls when a similar DA for ACP was evaluated in another study [51]. The DA on insurance plan selection neither improved financial toxicity nor reduced the proportion of participants who delayed or avoided care because of the insurance cost [49].

### Challenges of DA implementation

Regarding DA characteristics, the strengths of an electronic format for updating and dissemination [41] have been highlighted with concurrent concerns over the computer access and cancer survivors’ preferences for the paper format [46]. Additional challenges included limited users’ engagement in discussion forums and real-time connectivity to care providers, compounded by inadequate navigational support [52]. One study which explored healthcare professionals’ perspectives revealed low self-efficacy and a lack of perceived usefulness of the DA as implementation barriers [47].

Recurring determinants of successful DA implementation were characteristic of an individual cancer survivor. No consensus was reached regarding the appropriate timing to introduce DAs across studies, which depended on factors such as survivors’ emotions and time after diagnosis [41, 46, 48, 49, 52]. Other clinical and demographic factors, such as old age, poor health literacy, cognitive impairment, and low competence to forecast future preferences, were anticipated implementation challenges [42, 43]. Regarding the implementation of DAs for substance use among AYAs, the impact of the social environment—the behavior of AYAs’ family and friends—and the DA’s compatibility with school curricula were the anticipated implementation barriers [43]. A restricted range of health insurance plans applicable to cancer survivors affected the DA for insurance plan selection [49]. Two studies reported survivors’ preference for DA usage in the clinical care settings [41, 46].

## Discussion

To our best knowledge, this systematic review is the first to present the characteristics of the DAs that assist cancer survivors in deciding on survivorship care service engagement after completing primary treatment. The available DA post-treatment is underexamined contrary to treatment decisions but highlights ongoing efforts to identify complex decisions in the entire survivorship continuum. While the number of identified DAs was limited for each domain of the quality survivorship care framework, the range was comprehensive and addressed a spectrum of survivorship care needs. Included DAs were acceptable with favorable outcomes in improving knowledge despite inconclusive decisional outcomes.

The available DAs for the post-treatment phase suggested different decisional needs from the active treatment phase, shifting from disease management to wellness promotion [54]. This review highlighted healthcare delivery as a unique decisional aspect during survivorship. Klaassen et al. [46] developed a DA for specialist surveillance follow-up intensity, which reverses the trend of overusing specialist services during survivorship despite marginal benefit on mortality [55–57]. This review also identified two types of decisions considered during active treatment or previvor stage—fertility management and genetic testing [25, 58]. The DAs targeting the post-treatment phase underscored the challenges associated with extending the use of DAs developed for earlier phases. First, DA transferability was limited by the side effects of cancer treatment. For instance, family-building goals have shifted from a fertility preservation approach to assisted reproductive technology. Yet, unaddressed fertility information needs and decision distress were reported in post-treatment women of reproductive age, which compromised survivors’ quality of life and reinforced the value of decision support in this survivor population [59, 60]. Apart from the disparate options that affect transferability, the information needs may differ in quality. Specific to BRCA genetic testing, high-risk breast cancer survivors were keen on information related to the recurrence of the affected breast. Moreover, previvors were more interested in the impact of risk-reducing surgery [44]. The DA design for the post-treatment phase requires careful consideration of the residual treatment effects, available care options, and unique information for this phase.

The DAs’ specificity in the post-treatment survivorship phase should be counterbalanced with efficiency considerations. Among the included studies, DAs for ACP and insurance plan selection were applicable from the time of cancer diagnosis [42, 48, 49, 51–53]. The sustained ACP applicability throughout life and the integration of a personalized cost calculator are relevant despite non-specificity. Moreover, DAs addressing health promotion and disease prevention present opportunities for DA adaptations. One study modeled its DA after the validated Statin Choice DA [61], illustrating an attempt to adapt an evidence-based generic tool for cancer survivors by supplementing cardiovascular disease risk estimates specific to cancer treatment history. This approach can apply to other DAs for health promotion, such as smoking cessation, physical activity, and weight loss programs, evaluated in non-cancer populations [62–64]. Based on a wide range of survivorship care domains, developers could tap available DAs to improve efficiency and apply DAs from acute to extended survivorship and from the general population to the cancer population.

Our results accentuate that the AYA population is unique for decision support. Although only one DA for substance use targeted this age group, its unique content reinforced AYAs’ distinct characteristics [43]. AYA cancer survivors encounter survivorship care decisions while developing full autonomy, and parents may play a role in decision-making [65]. The additional health decision-making module in the DA for substance use aimed to improve AYAs’ self-efficacy, which strongly correlated with higher degrees of self-regulatory skills and perceived autonomy [66]. Furthermore, the innovative presentation via interactive pathways was compatible with AYAs’ preferred format of contextualizing information to their way of life, instead of a factual presentation format employed predominantly by DAs targeting the adult population [67]. DA development for this age group warrants consideration of additional strategies to promote self-efficacy and ensure compatibility of information with AYAs’ preference to improve communication.

Similar to the Cochrane systematic review on cancer screening and treatment decisions [20], studies that evaluated DAs for ACP also demonstrated improved knowledge of available care options [40, 49, 53]. However, non-significant effects on decisional outcomes observed in most studies have possible explanatory factors. First, the quality of most RCTs was compromised with a high risk of bias due to missing outcome data. Second, multiple pilot RCTs examining the feasibility of DA usage lacked statistical power for conclusive inference. Third, the impact of DAs may be short-lived relative to the behavioral change required when engaging with survivorship care services. Despite improved knowledge, DAs did not improve intentions to participate in screening or did not modify risky lifestyle behaviors [40, 43]. This observation could be attributed to survivors’ failure to cognitively associate such health behaviors with potential threats and disproportionate focus on the cancer condition since diagnosis [68]. One DA promoted a higher adoption of a less intensive follow-up modality among breast cancer survivors than controls, highlighting the usefulness of DAs for care model selection after primary treatment [46]. Apart from the range of outcomes evaluated, levels of self-efficacy and motivation are constructs of interest as behavioral change underlies survivorship care service engagement.

In this review, the anticipated DA implementation challenges were consistent with the system-level factors reported in the SDM literature [69, 70]. With a wide range of applicable DAs in the survivorship phase, a systematic approach to consolidating DA dissemination through a library catalog could facilitate comprehensive access. Since the content structure of most included DAs was similar, a recurring template could be employed. Additional interactive platforms should be considered to improve connectivity between the user and the survivor community or care providers. Additionally, DA usage should be personalized based on the survivor characteristics highlighted to affect DA uptake. Specifically, healthcare professionals should tailor DA to each survivor’s preferred format, readiness for change, literacy, age, and setting. Notably, for AYAs, engagement with family members or friends in the survivor’s relationship network is critical for promoting a conducive social environment to achieve behavioral change and decision-making [71, 72].

This review has several limitations. First, non-RCTs not ranked the highest on the hierarchy of evidence were included to capture an exhaustive range of DAs [73]. Second, the mixed quality of the included studies may affect the reliability of outcomes. Third, the generalizability of the results beyond the American and European populations to other regions with different cultures may be challenging, given that these countries have relatively better SDM awareness [74]. Notably, the concept of SDM underlying the utility of DAs may be foreign to survivors and clinicians from settings with a stronger hierarchical culture [75, 76].

## Conclusions

This systematic review identified a diverse range of DAs to support cancer survivors’ decisions over survivorship care service engagement after primary treatment. Despite inconclusive results on decisional outcomes, care settings can implement available DAs for knowledge improvement. Prospective DA developers should examine the need for cancer survivor specificity and explore if existing DAs available for the treatment phase or non-cancer populations could be readily adopted, especially for health promotion. Particularly, this review highlighted AYAs as a special population for tailored DA design and implementation considerations. Future work should clarify the impact of DAs on decisional outcomes or behavior-related constructs.

### Supplementary Information

Below is the link to the electronic supplementary material.

Supplementary file1 (DOCX 26 KB)

Supplementary file2 (DOCX 20 KB)

Supplementary file3 (DOCX 28 KB)

## Data Availability

All data generated or analyzed during this study are included in this published article and its supplementary information files.
