# Supplementary material for: Decision aids for cancer survivors’ engagement with survivorship care services after primary treatment: a systematic review
Source: J Cancer Surviv. 2022 Jul 7;18(2):288–317. doi: 10.1007/s11764-022-01230-y (PMC10960885; doi:10.1007/s11764-022-01230-y)
Supplement: Supplementary file 2 — Supplementary file2 (DOCX 20 KB) [file 11764_2022_1230_MOESM2_ESM.docx]

**Title**: Decision aids for cancer survivors’ engagement with survivorship care services after primary treatment: a systematic review

**Journal name**: Journal of Cancer Survivorship

**Authors**: Yu Ke, Hanzhang Zhou, Raymond Javan Chan, Alexandre Chan

**Corresponding author**:

Dr Alexandre Chan

Affiliation: University of California, Irvine

Email: [a.chan@uci.edu](mailto:a.chan@uci.edu)

**Supplementary File 2** Search Strategy

| **No.** | **Search Terms** | **Database** | **No. of returns** |
| --- | --- | --- | --- |
| 1 | 1. ((((neoplasms[mesh]) OR (cancer*)) AND ((((((survivors[mesh]) OR (patients[mesh])) OR (survivorship[mesh])) OR (survivor*)) OR (patient*)) OR (survivorship))) OR ((cancer survivors[mesh]) OR ("cancer survivors*"))) AND ((decision support techniques[mesh]) OR ("decision aid*")) 2. ((((neoplasms[mesh]) OR (cancer*)) AND ((((palliative care[mesh]) OR (palliative)) OR (supportive)) OR (post-treatment*))) AND ((decision support techniques[mesh]) OR ("decision aid*"))) 3. 1 OR 2 | PubMed | 8429 |
| 2 | 1. malignant neoplasm'/exp AND ('survivor'/exp OR 'survivorship'/exp OR 'patient'/exp) AND ('decision aid'/exp OR 'decision tool' OR 'decision support tool'/exp) AND ([english]/lim) 2. cancer survivor'/exp AND ('decision aid'/exp OR 'decision tool' OR 'decision support tool'/exp) AND ([english]/lim) 3. malignant neoplasm'/exp AND ('decision aid'/exp OR 'decision tool' OR 'decision support tool'/exp) AND (palliative OR 'supportive care'/exp OR 'post-treatment') AND ([english]/lim) 4. 1 OR 2 OR 3 | Embase | 124 |
| 6 | 1. exp Breast Neoplasms/ or exp Nervous System Neoplasms/ or exp Brain Neoplasms/ or exp Neoplasms/ or exp Endocrine Neoplasms/ or exp Benign Neoplasms/ or neoplasm*.mp. or cancer*.mp 2. survivor*.mp or patient*.mp or survivorship.mp 3. decision making/ or *choice behavior/ or exp Decision Support Systems/ or ((decision* or decid*) adj (support* or aid* or tool* or technique*)).mp 4. 1 and 2 and 3 5. limit 4 to (english language and yr="1860 - 2021") | PsycINFO | 2118 |
| 7 | 1. ( MH neoplasms+ AND cancer* ) AND ( ( MH survivorship+ OR MH survivors+ OR MH patients+ ) OR ( survivor* OR patient* OR survivorship ) ) AND ( MH "decision support techniques+" OR MH decision support systems, clinical OR ( ((decision* or decid*) and (support* or aid* or tool* or technique*)) ) ) 2. ( MH “cancer survivors+" OR "cancer survivor*" ) AND ( MH "decision support techniques+" OR MH decision support systems, clinical OR ( ((decision* or decid*) and (support* or aid* or tool* or technique*)) ) ) 3. 3. 19 OR 20 | CINAHL | 5577 |
