# Supplementary material for: Decision aids for cancer survivors’ engagement with survivorship care services after primary treatment: a systematic review
Source: J Cancer Surviv. 2022 Jul 7;18(2):288–317. doi: 10.1007/s11764-022-01230-y (PMC10960885; doi:10.1007/s11764-022-01230-y)
Supplement: Supplementary file 3 — Supplementary file3 (DOCX 28 KB) [file 11764_2022_1230_MOESM3_ESM.docx]

**Title**: Decision aids for cancer survivors’ engagement with survivorship care services after primary treatment: a systematic review

**Journal name**: Journal of Cancer Survivorship

**Authors**: Yu Ke, Hanzhang Zhou, Raymond Javan Chan, Alexandre Chan

**Corresponding author**:

Dr Alexandre Chan

Affiliation: University of California, Irvine

Email: [a.chan@uci.edu](mailto:a.chan@uci.edu)

**Supplementary File 3** Study Quality Assessment

Randomized trials – *version 2 of the Cochrane risk-of-bias tool for randomized trials (RoB 2)*

| **Study** | **Risk of bias arising from the randomization process** | **Risk of bias due to deviations from the intended interventions** | **Risk of bias due to missing outcome data** | **Risk of bias in measurement of the outcome** | **Risk of bias in selection of the reported result** | **Overall risk of bias** |
| --- | --- | --- | --- | --- | --- | --- |
| Hollen et al. (2013) | Some concerns | Low | Low | Low | Some concerns | Some concerns |
| Matlock et al. (2014) | Low | Low | High | High | Some concerns | High |
| Smith et al. (2020) | Low | Some concerns | High | Low | Some concerns | High |
| Vogel et al. (2013) | Low | Low | High | Low | Some concerns | High |
| Yun et al. (2019) | Low | Low | Low | High | Some concerns | High |

Non-randomized trials – *Risk Of Bias In Non-randomized Studies of Interventions (ROBINS-I)*

| **Study** | **Risk of bias due to confounding** | **Risk of bias in selection of participants into the study** | **Risk of bias in classification of interventions** | **Risk of bias due to deviations from intended interventions** | **Risk of bias due to missing data** | **Risk of bias in measurement of outcomes** | **Risk of bias in selection of the reported result** | **Overall risk of bias** |
| --- | --- | --- | --- | --- | --- | --- | --- | --- |
| Klaassen et al. (2018) | Low | Low | Low | Low | Moderate | Low | No information | Low |
| Politi et al. (2020) | Low | Low | Low | Low | Moderate | Serious | No information | Serious |

Cross-sectional studies – *Joanna Briggs Institute Checklist for Analytical Cross-Sectional Studies*

|  | **Green et al. (2009)** | **Klaassen et al. (2020)** |
| --- | --- | --- |
| 1. Were the criteria for inclusion in the sample clearly defined? | No | Yes |
| 2. Were the study subjects and the setting described in detail? | No | Yes |
| 3. Was the exposure measured in a valid and reliable way? | Yes | Yes |
| 4. Were objective, standard criteria used for measurement of the condition? | Unclear | Not applicable |
| 5. Were confounding factors identified? | Not applicable | Yes |
| 6. Were strategies to deal with confounding factors stated? | Not applicable | Not applicable |
| 7. Were the outcomes measured in a valid and reliable way? | Unclear | No |
| 8. Was appropriate statistical analysis used? | Unclear | Yes |

Qualitative studies/ component – *Critical Appraisal Skills Programme (CASP) Checklist*

|  | **Culver et al. (2011)** | **Kautz-Freimuth et al. (2021)** | **Klaassen et al. (2018)** |
| --- | --- | --- | --- |
| **Section A: Are the results valid?** | | | |
| 1. Was there a clear statement of the aims of the research? | Yes | Yes | Yes |
| 2. Is a qualitative methodology appropriate? | Yes | Yes | Yes |
| **Is it worth continuing?** | | | |
| 3. Was the research design appropriate to address the aims of the research? | Yes | Yes | Yes |
| 4. Was the recruitment strategy appropriate to the aims of the research? | Yes | Yes | Yes |
| 5. Was the data collected in a way that addressed the research issue? | Yes | Yes | Yes |
| 6. Has the relationship between researcher and participants been adequately considered? | Can’t tell | Can’t tell | Can’t tell |
| **Section B: What are the results?** | | | |
| 7. Have ethical issues been taken into consideration? | Yes | Yes | Yes |
| 8. Was the data analysis sufficiently rigorous? | Yes | Yes | Yes |
| 9. Is there a clear statement of findings? | Yes | Yes | Yes |
| **Section C: Will the results help locally?** | | | |
| **10.** How valuable is the research? | Understand decision making process among breast cancer survivors who are also BRCA mutation carriers, the study highlighted ways to revise decision aid for further effectiveness evaluation and implementation. | The study described the development of the first evidence-based, structured decision aids for previvors and survivors with BRCA mutations in Germany, representing an innovative addition to the range of specialised consulting services offered in in specialised care. | The needs and preferences identified in the study will have practical implications for the intended decision aid. |
| Abbreviation: BRCA, BReast CAncer gene. | | | |

Mixed methods studies – *Mixed Methods Appraisal Tool (MMAT) version 2018*

| **Category of study designs** | **Methodological quality criteria** | **Broadbent et al. (2021)** | **Raghunathan et al. (2020)** |
| --- | --- | --- | --- |
| Screening questions | S1. Are there clear research questions? | Yes | Yes |
|  | S2. Do the collected data allow to address the research questions? | Yes | Yes |
| 1. Qualitative | 1.1. Is the qualitative approach appropriate to answer the research question? | Yes | Yes |
|  | 1.2. Are the qualitative data collection methods adequate to address the research question? | Yes | Yes |
|  | 1.3. Are the findings adequately derived from the data? | Yes | No |
|  | 1.4. Is the interpretation of results sufficiently substantiated by data? | Yes | Yes |
|  | 1.5. Is there coherence between qualitative data sources, collection, analysis and interpretation? | Yes | Yes |
| 3. Quantitative non-randomized | 3.1. Are the participants representative of the target population? | Not applicable | Yes |
|  | 3.2. Are measurements appropriate regarding both the outcome and intervention (or exposure)? | Not applicable | Yes |
|  | 3.3. Are there complete outcome data? | Not applicable | Yes |
|  | 3.4. Are the confounders accounted for in the design and analysis? | Not applicable | Yes |
|  | 3.5. During the study period, is the intervention administered (or exposure occurred) as intended? | Not applicable | Yes |
| 4. Quantitative descriptive | 4.1. Is the sampling strategy relevant to address the research question? | No | Not applicable |
|  | 4.2. Is the sample representative of the target population? | No | Not applicable |
|  | 4.3. Are the measurements appropriate? | Yes | Not applicable |
|  | 4.4. Is the risk of nonresponse bias low? | Can’t tell | Not applicable |
|  | 4.5. Is the statistical analysis appropriate to answer the research question? | Yes | Not applicable |
| 5. Mixed methods | 5.1. Is there an adequate rationale for using a mixed methods design to address the research question? | Yes | No |
|  | 5.2. Are the different components of the study effectively integrated to answer the research question? | No | Yes |
|  | 5.3. Are the outputs of the integration of qualitative and quantitative components adequately interpreted? | No | Yes |
|  | 5.4. Are divergences and inconsistencies between quantitative and qualitative results adequately addressed? | No | Yes |
|  | 5.5. Do the different components of the study adhere to the quality criteria of each tradition of the methods involved? | No | Yes |
